# Analysis of Inbreeding, Population Structure, and Genetic Diversity in the Kumamoto Sub-Breed of Japanese Brown Cattle

**DOI:** 10.3390/ani16162587

**Published:** 2026-08-19

**Authors:** Tenghui Wang, Keiichi Inoue, Kasumi Ichinoseki, Masayuki Takeda, Yo Fukuzawa, Takatoshi Ozaki, Wei Peng, Guowen Wang, Takafumi Ishida

**Affiliations:** 1 Academy of Animal and Veterinary Sciences, Qinghai University, Xining 810016, China; 2 Faculty of Agriculture, University of Miyazaki, Miyazaki 889-2192, Japan; 3 National Livestock Breeding Center, Nishigo, Fukushima 961-8511, Japan; 4 Graduate School of Agricultural Science, Tohoku University, Sendai 980-8572, Japan; 5 Nihon Akaushi Registration Association, Kumamoto 861-2101, Japan

**Keywords:** small livestock population, Japanese Brown cattle, inbreeding coefficient, population structure, genetic diversity, sire background, conservation priority

## Abstract

**Introduction:** The Kumamoto sub-breed of Japanese Brown cattle is a small population facing increasing inbreeding and declining effective population size driven by the intensive use of a limited number of elite sires. **Methods:** We analyzed 811 Japanese Brown cows genotyped using a 30K SNP array, retaining 19,745 SNPs after quality control. We calculated and compared ten genomic inbreeding estimators, including SNP-by-SNP and segment-based measures. Population structure was first assessed by rearing region using principal component analysis (PCA) and was then evaluated using ADMIXTURE-based clustering and distance-based hierarchical clustering, from which representative subsets were retained under predefined filtering criteria and further examined using PCA and neighbor-joining (NJ) tree analyses. Finally, we calculated the contribution of each ADMIXTURE group to total gene and allelic diversity, and integrated these two measures into a final conservation index after Z-score standardization. **Results:** ROH- and HBD-based estimators showed high concordance, whereas allele-frequency-dependent SNP-by-SNP estimators exhibited distinct distributions. Rearing region did not explain the main genetic structure. Instead, four representative ADMIXTURE-based groups, supported by independently identified family groups, captured the major genetic structure associated with paternal backgrounds. ADMIXTURE group 2 made the largest contribution to both gene and allelic diversity, and showed lowest genomic inbreeding. **Conclusions:** These findings suggest that ROH- and HBD-based estimators may serve as valuable indicators of genomic inbreeding in the Kumamoto sub-breed of Japanese Brown cattle; paternal background has played an important role in shaping the current genomic structure; and ADMIXTURE group 2, mainly associated with the Haru-yama-to/-sakae sire background, may provide a valuable breeding resource for limiting future inbreeding accumulation and maintaining genetic diversity.

## Simple Summary

The Kumamoto sub-breed of Japanese Brown cattle is a local beef cattle population in Japan. It is facing increasing inbreeding and a declining effective population size because of the intensive use of a limited number of elite sires. This study used genetic marker information from 811 cows to evaluate inbreeding, describe the genetic structure of the population, and identify genetic subpopulations that may help to preserve genetic diversity. We found that segment-based measures gave consistent information about inbreeding. The main genetic differences among animals were not explained by where they were raised, but were closely related to their paternal background. Among the identified subpopulations, we found that the subpopulation mainly associated with the Haru-yama-to/-sakae sire background contributed most to overall genetic diversity and showed the lowest level of genomic inbreeding. These results suggest that individuals from this subpopulation may be useful for breeding designing aimed at controlling inbreeding and maintaining genetic diversity in the Kumamoto sub-breed.

## 1. Introduction

Japanese Brown cattle is one of the Japanese Wagyu breeds and comprises two sub-breeds, Kumamoto and Koichi [1]. The Kumamoto sub-breed originated from cattle introduced from the Korean Peninsula centuries ago and underwent a systematic improvement through crossbreeding with imported breeds, particularly Simmental, beginning around 1900; it was officially recognized as one of Wagyu breeds (Japanese Brown) in 1944. Primarily reared in Kumamoto Prefecture, this sub-breed accounts for most of the Japanese Brown population, with approximately 21,450 animals. Its distinctive characteristics include a light-brown coat, good grazing adaptability, and favorable growth performance. It also produces beef with moderate marbling, a trait increasingly favored by Japanese consumers. In recent decades, the expanding use of reproductive technologies, especially artificial insemination (AI), combined with modern genetic evaluation and selection decisions, has significantly accelerated genetic progress for economically important traits in livestock populations, including Japanese Brown cattle [2,3,4]. Sumio [5] reported that standardized estimated transmitting ability (ETA) trends in Japanese Brown cattle showed a marked increase in Beef Marbling Standard number (BMS No.), indicating sustained genetic improvement in marbling without an apparent decline in major carcass traits. However, this technology-driven process has inevitably reshaped population structure by facilitating the rapid dissemination of a small number of elite sires, leading to the dominance of few paternal lineages [6,7]. According to statistics published by the Japan Akaushi Registration Association in 2007, breeding females in Kumamoto Prefecture were disproportionately derived from two paternal lineages, Mitsu-take and Shige-nami, which together accounted for more than 90% of breeding females. In addition, as genetic improvement is current pursued only within the existing wagyu gene pool and the breeding population continues to decline [8,9], the scope for broadening the genetic base in Japanese Brown cattle is constrained, thereby intensifying inbreeding accumulation and increasing the risk of inbreeding depression. As reported by Honda et al. [10], the effective population size of Japanese Brown cattle had continuously declined to 25.5 in the Kumamoto sub-breed and 6.0 in the Kochi sub-breed. The corresponding average inbreeding coefficients reached 7.1% and 8.8%, values that were numerically higher than the 6.25% individual-level benchmark commonly used in pedigree beef cattle breeding [11]. A comprehensive assessment of inbreeding, population structure and genetic diversity in this numerically small breed is therefore increasingly important for future breeding programs and long-term genetic improvement.

Inbreeding refers to the mating of related individuals, resulting in increased homozygosity and reduced genetic diversity in their offspring [12]. Increased homozygosity can expose deleterious recessive alleles in natural populations and reduce the frequency of advantageous heterozygous genotypes at loci showing overdominance. These changes can decrease body size, vigor, and reproductive fitness, a phenomenon known as inbreeding depression [13,14]. The inbreeding coefficient is a key parameter for studying inbreeding depression and managing populations under conservation [15]. Wright [16] first defined the inbreeding coefficient as the correlation between homologous alleles in the two gametes forming an individual, whereas later the inbreeding coefficient was interpreted as the probability that the two alleles at a locus are identical by descent [17]. Traditionally, the inbreeding coefficient have been estimated from pedigree records, although the utility of pedigree-based estimates is often limited by shallow pedigree depth, incomplete records, and uncontrolled mating [18]. In recent years, the growing availability of genomic information, especially single-nucleotide polymorphism (SNP) data, has promoted the widespread use of genomic inbreeding coefficients to assess inbreeding levels in livestock populations, even when pedigree information is unavailable. By capturing realized homozygosity, genomic estimators can provide more accurate estimates of individual inbreeding than pedigree-based measures [19]. Genomic inbreeding coefficients can be broadly divided into two methodological groups [6]: (1) those based on individual SNPs (SNP-by-SNP estimators), which rely on allele frequencies in the base population, and (2) those based on chromosome segments, which may provide useful information for distinguishing autozygosity from homozygosity and for inferring recent versus ancient inbreeding. Their performance is affected by factors such as sensitivity to recent versus ancient inbreeding, dependence on allele frequency assumptions, marker density, and effective population size [17,20]. Hence, there is still no consensus on which estimator is universally optimal.

Population genetic structure, defined as the non-random distribution of genetic variation within and between populations [21,22], is another important aspect in population management. It can reflect demographic processes such as isolation and divergence, population bottlenecks, or admixture [23,24]. In practice, population structure analyses often start from some predefined classifications based on subjective labels, such as linguistic, cultural, physical characters, or geographic information [25]. Although this approach is usually sensible but may fail to detect cryptic population structure, which can bias downstream analyses, such as genome-wide association studies [26], genomic prediction [27] and breeding decisions [28]. Advances in genome sequencing technology may open up new possibilities for measuring population genetic structure with greater precision [29]. Genomic information enables genetic clustering of individuals through distance-based [30], multivariate model-free [31], and model-based approaches [32], allowing us to investigate the relationship between these clusters and predefined labels.

In addition, understanding genetic diversity in livestock is essential for developing effective management and conservation strategies, particularly for small and potentially vulnerable populations. So far, several genomic studies have investigated inbreeding level, inbreeding depression, genetic structure and diversity in Japanese Black cattle [33,34,35,36]. To our best knowledge, however, this is not yet the case in Japanese Brown cattle.

In this study, to provide a comprehensive characterization of inbreeding, genetic structure, and genetic diversity in the Kumamoto sub-breed of Japanese Brown cattle, we calculated and compared ten commonly used genomic inbreeding coefficients, characterized population structure through principal component analysis (PCA), model-based and distance-based clustering approaches and evaluated the contributions of representative subpopulations selected using predefined filters to overall genetic diversity.

## 2. Materials and Methods

### 2.1. Animals and Genotypes

Ethical approval was not required for this study because all data were obtained from the existing database of the National Livestock Breeding Center (NLBC), Japan.

This study comprised 811 Japanese Brown cows born between 2021 and 2023, sampled from farms in Kumamoto Prefecture (*n* = 754), Nagasaki Prefecture (*n* = 20), and Hokkaido Prefecture (*n* = 37). Because Kumamoto sub-breed of Japanese Brown cattle are mainly reared in Kumamoto Prefecture, the individuals from Kumamoto Prefecture were further divided into nine rearing-region groups according to local cattle-management units, whereas animals from Hokkaido and Nagasaki were each treated as a single group. The number of animals sampled from each rearing region was determined with reference to the number of Japanese Brown cattle reared within the corresponding management unit. The geographical distribution of the animals and the sample size of each rearing-region group are summarized in Figure S1.

Genotype data were obtained from the database of NLBC, Japan. All animals were genotyped using the GeneSeek Genomic Profiler (GGP) BovineLD v4.0 array, which contains 30,105 SNPs (Illumina, San Diego, CA, USA). All SNPs were filtered for call rate < 0.95, minor allele frequency (MAF) < 0.01, and extreme deviation from Hardy–Weinberg equilibrium (*p* < 0.0001). After quality control, 811 genotyped animals and 19,745 SNPs remained in the final dataset.

### 2.2. Genomic Inbreeding Coefficient Analysis

We evaluated genomic inbreeding using ten estimators, including five SNP-by-SNP estimators (*F*_GRM1_, *F*_GRM2_, *F*_GRM05_, *F*_HOM_, and *F*_UNI_) and five segment-based estimators (*F*_ROH_, *F*_ROH15_, *F*_ROH30_, *F*_HBD_6R_ and *F*_HBD_7R_).

*F*_GRM1_ and *F*_GRM2_ were calculated from the diagonal elements of the genomic relationship matrix (GRM) constructed according to VanRaden’s first and second methods, respectively [37]. The matrix-based formulas for the diagonal elements can be equivalently rewritten as follow:

(1) $$F_{\mathrm{GRM}1}=\frac{\sum_{k=1}^{n}{\left(x_{k}-2p_{k}\right)}^{2}}{2\sum_{k=1}^{n}p_{k}(1-p_{k})}-1$$

(2) $$F_{\mathrm{GRM}2}=\frac{1}{n}\sum_{k=1}^{n}\frac{{\left(x_{k}-2p_{k}\right)}^{2}}{2p_{k}\left(1-p_{k}\right)}-1$$

where *n* is the total number of SNPs, *x_k_* is the genotype of an individual at SNP *k*, coded as 0, 1 or 2 for genotypes carrying zero, one, or two copies of the reference allele, respectively, and *p_k_* is the frequency of the reference allele in the base population. Both *F*_GRM1_ and *F*_GRM2_ can be interpreted as variance-standardized relationships minus one. The main difference between them lies in how marker information is combined across loci. *F*_GRM1_ is based on a ratio of sums, whereas *F*_GRM2_ is closer to a sum of ratios because markers are weighted by the reciprocal of their expected variance. Since observed allele frequencies rather than founder-population frequencies were used in this study, we additionally calculated a modified version of *F*_GRM1_ by setting all allele frequencies at 0.5 to address this limitation, and referred to it as *F*_GRM05_.

The *F*_GRM1_ and *F*_GRM05_ were calculated by our own program coded in the R language v4.5.2, and *F*_GRM2_ was obtained from the *F*_hat1_ output of the PLINK *--ibc* function [38].

Consistent with Wright’s definition of the inbreeding coefficient [16], *F*_UNI_ was calculated from the correlation between uniting gametes following Yang et al. [39] as follows:

(3) $$F_{UNI}=\frac{1}{n}\sum_{k=1}^{n}\frac{x_{k}^{2}-\left(1+2p_{k}\right)x_{k}+2p_{k}^{2}}{2p_{k}\left(1-p_{k}\right)}$$

The estimator *F*_HOM_, which reflects both the loss and gain of genetic diversity, was calculated from the ratio of the observed to the expected number of homozygous genotypes [38], as follows:

(4) $$F_{HOM}=1-\frac{1}{n}\sum_{k=1}^{n}\frac{x_{k}\left(2-x_{k}\right)}{2p_{k}\left(1-p_{k}\right)}$$

Thus, *F*_UNI_ can be interpreted as the Pearson correlation coefficient between the two uniting gametes, with allelic states at SNP loci treated as paired observations.

Both *F*_HOM_ and *F*_UNI_ were obtained from the PLINK *--ibc* output and correspond to *F*_hat2_ and *F*_hat3_, respectively.

Runs of homozygosity (ROH) are continuous homozygous chromosomal segments generated by the inheritance of identical haplotypes from a common ancestor [40]. Inbreeding gives rise to such autozygous segments with longer ROH generally reflecting more recent common ancestry [41]. The ROH-based inbreeding coefficient, *F*_ROH_, was calculated as the total length of all ROH divided by the total autosomal genome length covered by SNP markers. However, ROH can also occur in outbred populations and short ROH are difficult to detect reliably with low-density SNP arrays [42,43]. Therefore, appropriate thresholds are required to ensure that identified segments are more likely to reflect true autozygosity. In this study, ROH detection and *F*_ROH_ calculation were performed using the sliding window method implemented in the R package detectRUNS v0.9.6. To reduce spurious ROH detection, the following parameters and thresholds were applied: (i) a maximum of one opposite SNP was allowed in each scanning window, (ii) a maximum of one missing SNP was allowed in each scanning window, (iii) the scanning window size was 15 SNPs, (iv) the scanning window threshold was 0.05. (v) the minimum number of consecutive homozygous SNPs included in an ROH (*L*) was 46, (vi) the minimum ROH length was 1 Mbp, (vii) the minimum SNP density was 1 SNP per 200 kbp, and (viii) the maximum allowed gap between consecutive SNPs was 1 Mbp. To minimize the number of ROH occurring by chance, *L* was determined according to the formula proposed by Lencz et al. [44], as follows:

(5) $$L=\frac{\ln\left(\frac{\alpha }{n_{s}n_{i}}\right)}{\ln\left(1-het\right)}$$

where *α* is the proportion of false-positive ROH (set to 0.05), *n_s_* is the number of genotyped SNPs per individual, *n_i_* is the number of genotyped individuals, and *het* is the mean expected heterozygosity across all SNPs. To avoid underestimating *F*_ROH_ due to a stringent *L* setting, which may exclude short ROH segments reflecting inbreeding from more distant common ancestors, we additionally calculated two alternative estimators by setting *L* to 30 and 15, denoted as *F*_ROH_30_ and *F*_ROH_15_, respectively.

Homozygosity by descent (HBD), also known as autozygosity, represents the within-individual manifestation of identity by descent (IBD) at the genomic level [40]. Because HBD status at a given locus cannot be observed directly from genotype data, we used the hidden Markov model (HMM)-based multiple-HBD-class approach implemented in the R package RZooRoH v0.4.1 to infer their HBD states and estimate an HBD-based inbreeding coefficient, *F*_HBD_, following Druet & Gautier [45,46]. In this model, the genome of an individual was modeled as mosaics of segments from multiple HBD classes, representing ancestors from different past generations. Each class was characterized by a class-specific rate parameter *R_C_* that determines the expected segment length under an exponential distribution. Genome-wide realized inbreeding was estimated as the mean across loci of the posterior probability that a locus belongs to any HBD class, inferred using the forward-backward algorithm. In this study, the Mix6R and Mix7R models were used to calculate *F*_HBD_6R_ and *F*_HBD_7R_, respectively.

### 2.3. Comparison Among Inbreeding Estimators

Summary statistics were calculated, and the distributions of the ten inbreeding estimators were inspected. Pairwise Pearson correlation coefficients were calculated to assess the strength of relationship among all estimators. PCA was performed on the ten estimators using the prcomp function in R, and the results were visualized using the R package factoextra v2.0.0.

### 2.4. Population Structure Analysis

PCA was first performed using GCTA v1.95.1 to evaluate whether rearing region was a major factor shaping the current population structure of Japanese Brown cattle. The analysis was based on a GRM constructed from the linkage disequilibrium (LD)-pruned SNP dataset (*r*^2^ > 0.8). Individuals were grouped according to rearing region, and the first two principal components (PCs) were visualized using the ggplot2 package v4.0.3 in R.

Population genetic structure was inferred by ADMIXTURE v1.3.0 using LD-pruned SNP dataset (*r*^2^ > 0.8). ADMIXTURE can characterizes population structure by modeling each sample as a set of fractional assignments to a specified number (K) of clusters inferred using an unsupervised algorithm, with each cluster represented by a centroid in the space of variant allele frequencies [32,47,48]. By avoiding reliance on predefined population labels, ADMIXTURE can better reflect the spectrum of genetic structure across samples [49]. Fivefold cross-validation was conducted for K values from 2 to 20. Because the cross-validation error decreased continuously as K increased and no clear optimum was observed within a reasonable range (Table S1), the final value of K was guided by known paternal pedigree information [50]. Accordingly, K = 4 was chosen, consistent with the number of major sire founders. To describe the major genomic patterns and obtain representative subsets for downstream comparisons, individuals were classified according to the ADMIXTURE results at K = 4. Individuals were assigned to the group corresponding to their predominant ancestry component only when the highest ancestry proportion was >50% and the second-highest ancestry proportion was <25%; all others were treated as having ambiguous backgrounds and were excluded from subsequent ADMIXTURE group-based analyses.

To further investigate population structure, we performed distance-based clustering on the same LD-pruned SNP dataset to identify genomic family groups, following the general concept of previous study [51,52,53]. Pairwise IBD coefficients were estimated using the *--genome* command in PLINK v1.90 and PI_HAT value from the output were used as a measure of genetic similarity between individuals. A distance matrix was constructed from PI_HAT value as D=1−PI_HAT, where smaller values indicate closer genetic relationships. Hierarchical clustering was then performed in R using the complete linkage method. Clusters were defined using the hybrid method implemented in R package dynamicTreeCut v1.63-1, with cutHeight = 0.90, deepSplit = 2 and minClusterSize = 30; all other parameters were kept at their default settings. Individuals not stably assigned to any cluster were labeled as unassigned.

Core genomic family groups were selected sequentially. Candidate groups with a mean within-group PI_HAT > 0.1 were first retained. Among these candidates, between-family differentiation was evaluated using mean between-family PI_HAT thresholds of 0.06, 0.07, 0.08, 0.09, and 0.10. At each threshold, we retained the group combination for which all pairwise between-group mean PI_HAT values were below the threshold and the total number of individuals was maximized.

Genetic relationships were further evaluated by PCA and Neighbor-joining (NJ) tree analysis under two grouping schemes: ADMIXTURE groups and genomic family groups. For NJ tree construction, pairwise Nei’s genetic distances among individuals were calculated from the LD-pruned SNP dataset using the stamppNeisD function in the R package StAMPP v1.6.3, followed by tree inference with the nj function in the R package ape v5.8-1 and visualization using the iTOL v7.6 online platform [54].

### 2.5. Analysis of Contribution to Genetic Diversity

To characterize allele-frequency variation among the four ADMIXTURE groups, within-group MAFs were calculated from the LD-pruned dataset (*r*^2^ > 0.8) using PLINK v1.90. The MAF distribution was summarized into the following bins: rare alleles (0 < MAF ≤ 0.1), intermediate-frequency alleles (0.1 < MAF ≤ 0.2, 0.2 < MAF ≤ 0.3, and 0.3 < MAF ≤ 0.4), and common alleles (0.4 < MAF ≤ 0.5).

The contributions of each ADMIXTURE group to gene diversity and allelic diversity was calculated using metapop2 software v2.5.3 [55]. This analysis followed the methods proposed by Caballero & Toro [56] and Caballero & Rodríguez-Ramilo [57], in which total diversity of a metapopulation is partitioned into within- and between-subpopulation components. Here, each ADMIXTURE group was treated as a subpopulation within the metapopulation. The contribution of each group was evaluated as the percentage gain or loss in gene diversity and allelic diversity after removing that group from the metapopulation.

For the calculation of gene diversity, each subpopulation was weighted in proportion to its sample size. For a given locus, the mean frequency of allele *k* across *n* subpopulations was calculated as ${\bar{p}}_{k}=\frac{1}{n}\sum_{i=1}^{n}p_{i,k}$ and the total gene diversity $H_{T}$ was then calculated as $H_{T}=1-\sum_{k=1}^{K}{\bar{p}}_{k}^{2}$, where *k* denotes total number of alleles at the locus. The partition of $H_{T}$ followed:

(6) $$H_{T}=H_{S}+D_{G}=(1-\tilde{f})+D_{G}$$

where $H_{S}$ is the within-subpopulation component of gene diversity, $D_{G}$ is the between-subpopulation component, and $\tilde{f}$ denotes the average within-subpopulation coancestry.

For allelic diversity, the within-subpopulation allelic diversity of subpopulation *i*, denoted as $A_{s,i}$, was estimated using rarefaction to account for differences in sample size among subpopulations. The total allelic diversity can be partitioned into the average within-subpopulation allelic diversity $A_{S}$ and the average between-subpopulation allelic diversity $D_{A}$ as follow:

(7) $$A_{T}=A_{S}+D_{A}=\frac{1}{n}\sum_{i=1}^{n}\left(A_{S,i}+\frac{1}{n}\sum_{j=1}^{n}d_{A,ij}\right)$$

where $d_{A,ij}$ denotes the allelic distance between subpopulations *i* and *j*, defined as the average number of alleles present in one subpopulation but absent from the other. It should be noted that the diversity measures described above were calculated separately for each locus; in the multilocus case, the corresponding estimates were averaged over loci.

A synthetic pool (N = 1000) was constructed to estimate the optimal proportion of each subpopulation for maximizing total gene and allelic diversity. To enable comparisons across subpopulations, two sets of Z-score standardization were performed: one based on loss-or-gain contribution estimates and the other based on the optimal synthetic-pool proportions. Within each set, gene diversity and allelic diversity were standardized separately and then summed to obtain a final composite Z-score [58].

## 3. Results

### 3.1. Patterns of ROH Segments

**Figure 1:**
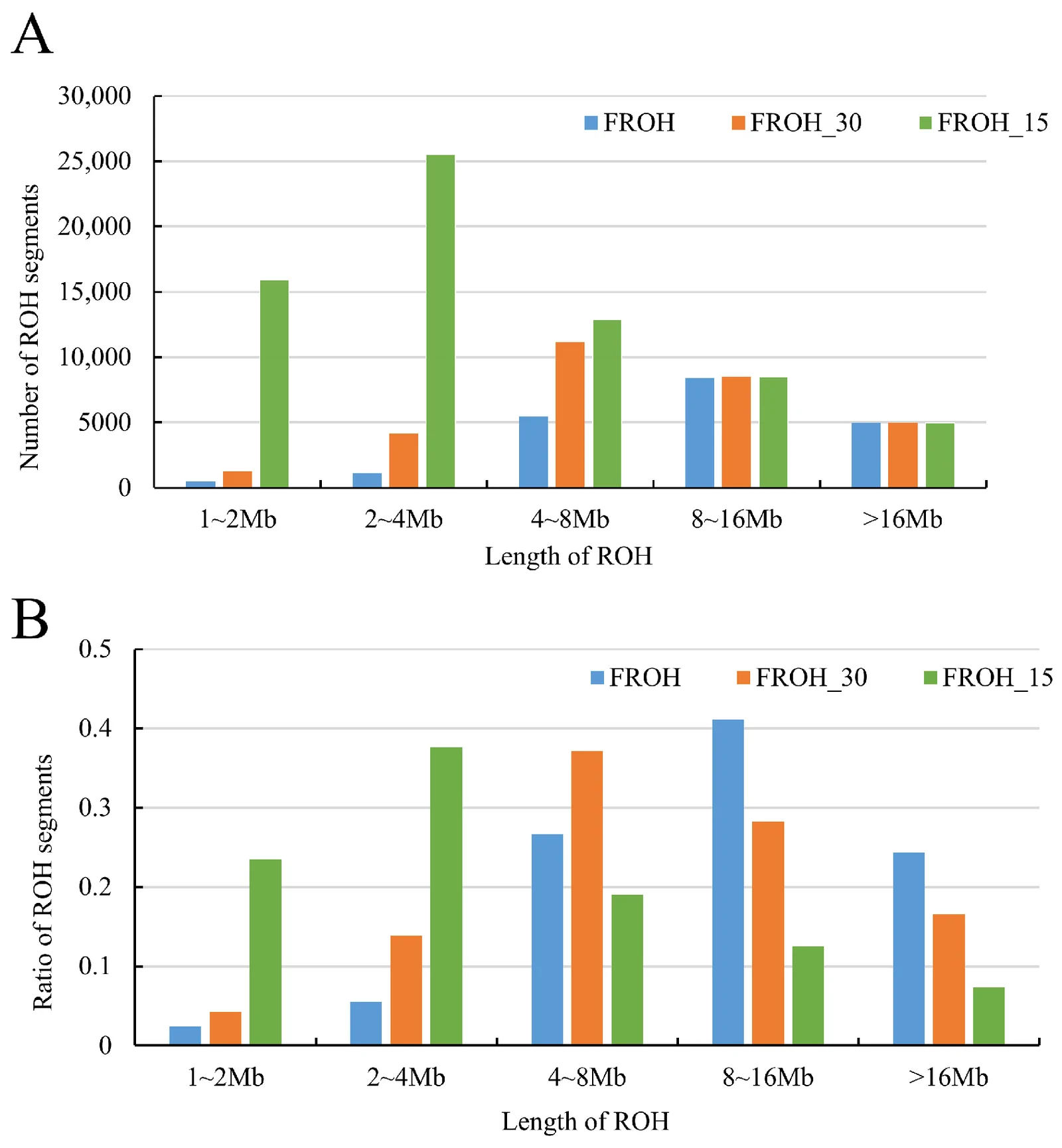
Distribution of the number (**A**) and proportion (**B**) of ROH segments under different minimum numbers of consecutive homozygous SNPs (*L*) included in ROH: 46 (*F*_ROH_), 30 (*F*_ROH_30_), and 15 (*F*_ROH_15_).

The distributions of the number and proportion of ROH segments are shown in Figure 1. The total numbers of ROH segments detected using *F*_ROH_, *F*_ROH_30_, and *F*_ROH_15_ were 20,461, 30,054, and 67,819, respectively. The numbers of ROH segments longer than 8 Mb were same among the three ROH-based inbreeding coefficients, whereas the numbers of ROH segments shorter than 4 Mb increased substantially with the reduction in *L*. Consequently, *F*_ROH_30_ and *F*_ROH_15_ showed higher proportions of short ROH segments than *F*_ROH_.

### 3.2. Comparison of Inbreeding Coefficients

**Figure 2:**
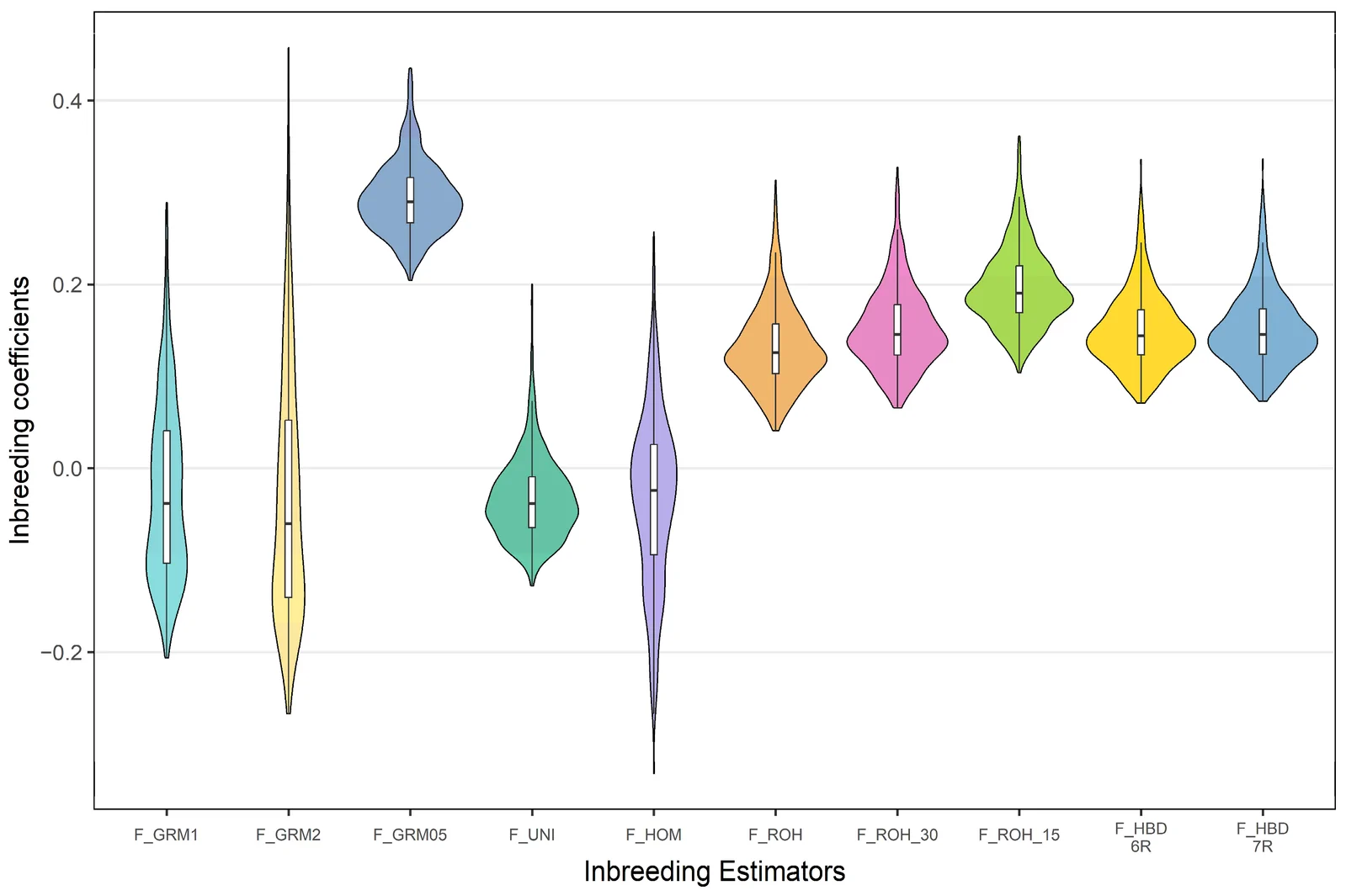
Violin plots of inbreeding coefficients calculated using ten estimators in the Japanese Brown cattle population. Colors indicate different estimators. The embedded boxplots show the median and interquartile range for each estimator.

Summary statistics for the ten inbreeding coefficients are shown in Table 1, and their distributions are shown in Figure 2. The ranges of the GRM-based inbreeding coefficients differed markedly: *F*_GRM1_ ranged from –0.206 to 0.289, *F*_GRM2_ from −0.267 to 0.457, and *F*_GRM05_ from 0.204 to 0.435. Among all estimators, *F*_GRM05_ yielded the highest overall estimates, with a mean of 0.294 and a median of 0.290. The ROH-based inbreeding coefficients ranged from 0.041 to 0.313 for *F*_ROH_, from 0.066 to 0.327 for *F*_ROH_30_, and from 0.104 to 0.361 for *F*_ROH_15_, showing an increasing trend as *L* decreased. The HBD-based estimators provided relatively stable estimates across model settings, with *F*_HBD_6R_ ranging from 0.071 to 0.336 and *F*_HBD_7R_ from 0.073 to 0.336, along with very similar means and medians. Among all estimators, negative values were observed for *F*_GRM1_, *F*_GRM2_, *F*_HOM_, and *F*_UNI_, with the first three showing relatively more dispersed distributions.

**Figure 3:**
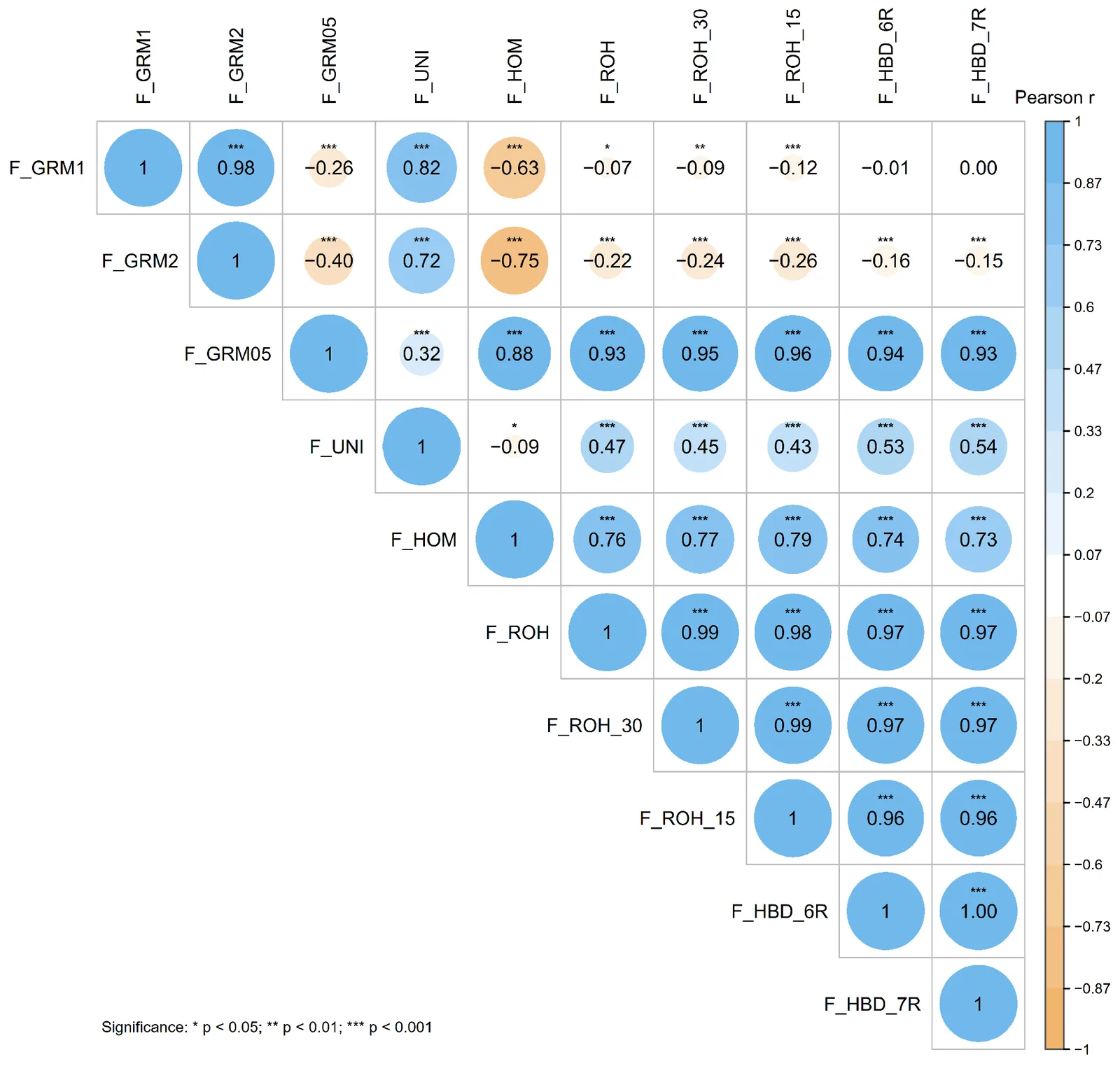
Pairwise Pearson correlations among the ten inbreeding estimators.

Pairwise Pearson correlation analysis revealed distinct relationships among the ten inbreeding estimators (Figure 3). *F*_GRM1_ and *F*_GRM2_ were strongly correlated with each other (r = 0.98), and both were negatively correlated with all other estimators except *F*_UNI_ (r = 0.82 and r = 0.72, respectively), including *F*_GRM05_ (r = −0.26 and r = −0.40, respectively). *F*_GRM05_ and *F*_HOM_ (r = 0.88) exhibited relatively high correlations with most estimators, with coefficients ranging from 0.93 to 0.96 for *F*_GRM05_ and from 0.73 to 0.79 for *F*_HOM_, except with *F*_GRM1_, *F*_GRM2_, and *F*_UNI_. The ROH- and HBD-based estimators were highly correlated with one another, ranging from 0.96 to 0.99; *F*_HBD_6R_ and *F*_HBD_7R_ were perfectly correlated (r = 1.00).

**Figure 4:**
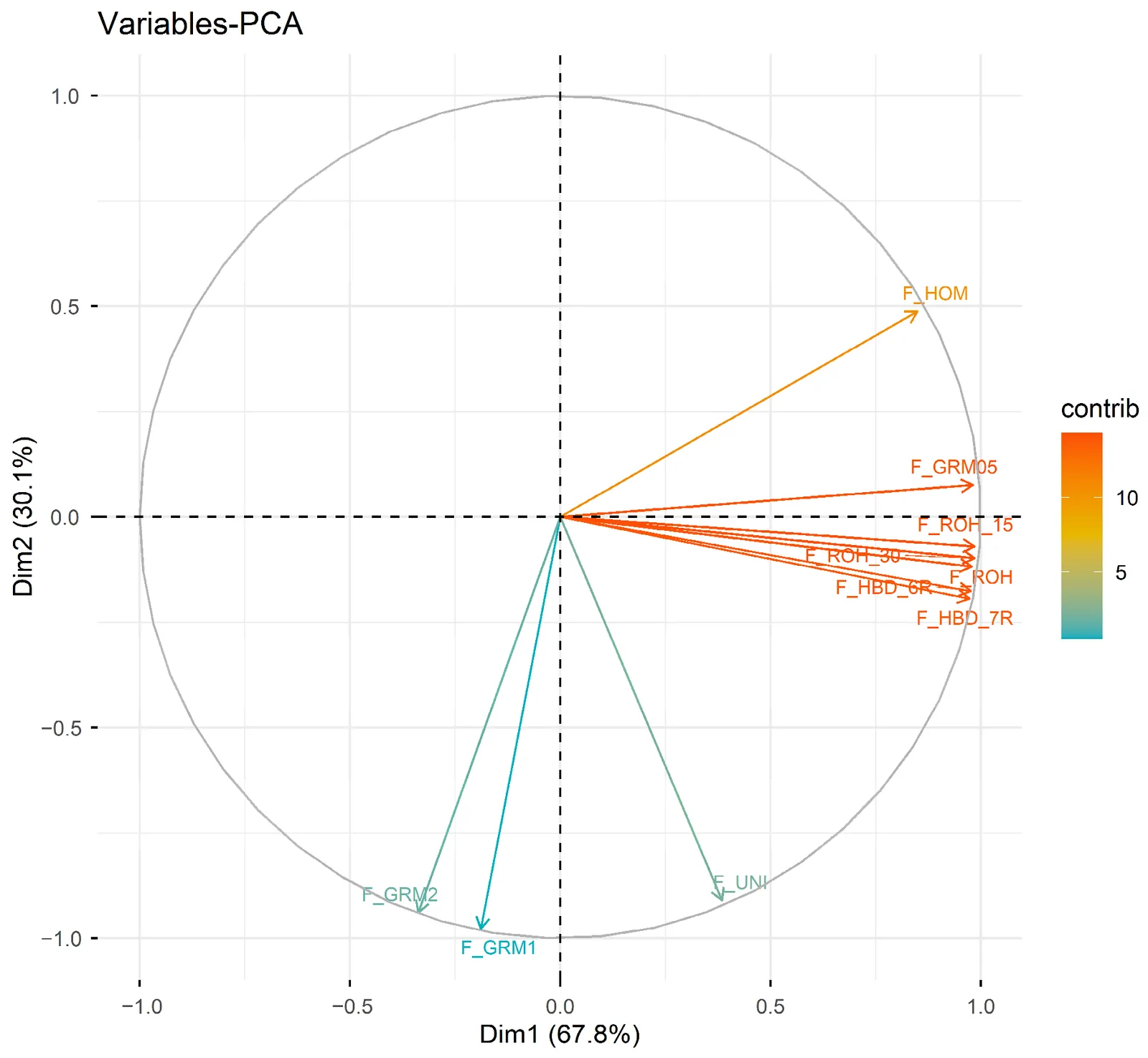
PCA of the ten inbreeding coefficient estimators. Scatterplot of variable loadings on the first two principal components (Dim1 and Dim2), with the percentage of variance explained by each component shown in parentheses. The plot was generated using the R package factoextra, and the color scale indicates the contribution (%) of each estimator on the first principal component, based on the squared cosine (cos2) quality of representation of the variables. The angles between arrows reflect correlations among estimators, with smaller angles indicating positive correlations and opposite directions indicating negative correlations. See Table 1 and the Materials and Methods section for detailed descriptions of the estimators.

The PCA results based on the ten inbreeding coefficient estimators are shown in Figure 4. The first two PCs explained most of the total variability, accounting for 97.8% in total (PC1, 67.8%; PC2, 30.1%). PC1 mainly separated *F*_GRM1_, *F*_GRM2_, and *F*_UNI_ from the remaining estimators. The ROH- and HBD-based estimators clustered closely together, indicating a high degree of similarity among these segment-based measures. PC2 mainly separated *F*_HOM_ from the other estimators, whereas *F*_GRM05_ was positioned close to the tightly clustered ROH- and HBD-based estimators.

### 3.3. Population Genetic Structure

**Figure 5:**
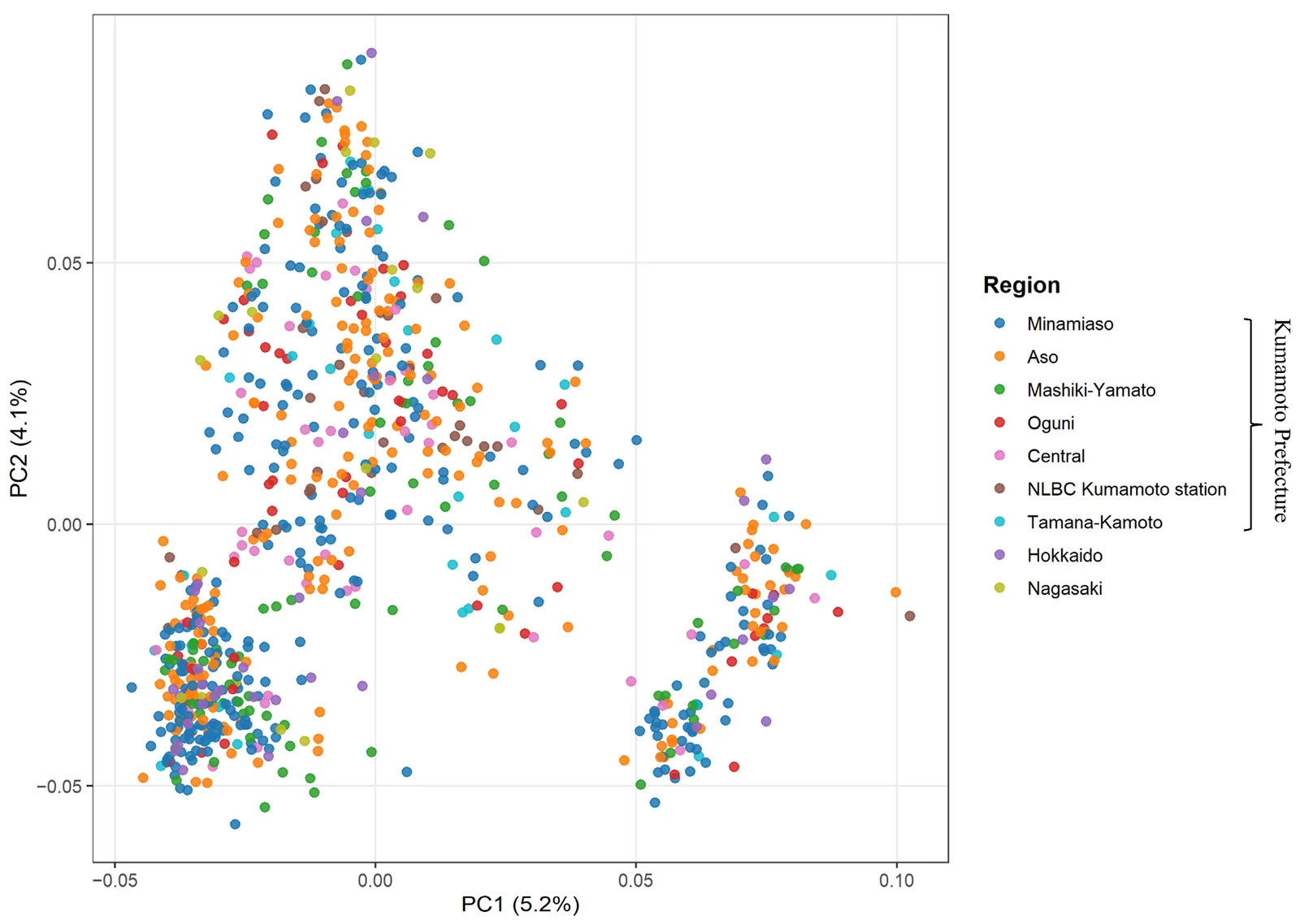
PCA of individuals grouped by rearing region.

For the PCA based on rearing regions, 11 individuals from two sampling sources were excluded due to small sample sizes, including nine from the Kumamoto Prefectural Livestock Research Center and two from Kuma in Kumamoto Prefecture. The remaining 800 individuals were assigned to nine rearing-region groups. Except for Hokkaido (*n* = 37) and Nagasaki (*n* = 20), all groups were located in Kumamoto Prefecture: Minamiaso (*n* = 280), Aso (*n* = 205), Mashiki-Yamato (*n* = 84), Oguni (*n* = 56), Central (*n* = 49), NLBC Kumamoto station (*n* = 35), and Tamana-Kamoto (*n* = 34). The PCA showed no clear differentiation among groups defined by rearing region (Figure 5).

Based on the ADMIXTURE results, individuals with clear predominant ancestry proportion were assigned to four genetic groups, hereafter referred to as ADMIXTURE groups (Table 2). Of the 811 individuals, 382 did not meet the assignment criteria and were therefore classified as unassigned. ADMIXTURE groups 1–4 contained 228, 52, 71, and 78 individuals, respectively.

Based on the pairwise IBD estimates, a PI_HAT-derived distance matrix was constructed as D = 1 − PI_HAT. Hierarchical clustering followed by tree cutting identified 10 candidate genomic family groups with at least 30 individuals (Table S2). The candidate groups ranged in size from 30 to 79 individuals, and all groups showed a mean within-group PI_HAT > 0.1. With increasingly stringent between-family mean PI_HAT thresholds from 0.10 to 0.06, the number of retained genomic family groups decreased from 6 to 4, and the number of retained individuals also decreased from 279 to 156 (Table 3). In all cases, the maximum between-group mean PI_HAT remained below the corresponding threshold. We treated the four family groups detected under the most stringent between-family mean PI_HAT threshold (0.06) as core genomic families. These core families comprised 156 individuals in total: Family 2 (*n* = 54), Family 6 (*n* = 39), Family 8 (*n* = 33), and Family 9 (*n* = 30) (Table 4). In the within- and between-family mean PI_HAT matrix, all pairwise between-family mean PI_HAT values were below 0.06, whereas the within-family mean PI_HAT values ranged from 0.243 to 0.318 (Table 5).

### 3.4. Assessment of ADMIXTURE Groups and Core Family Groups

**Figure 6:**
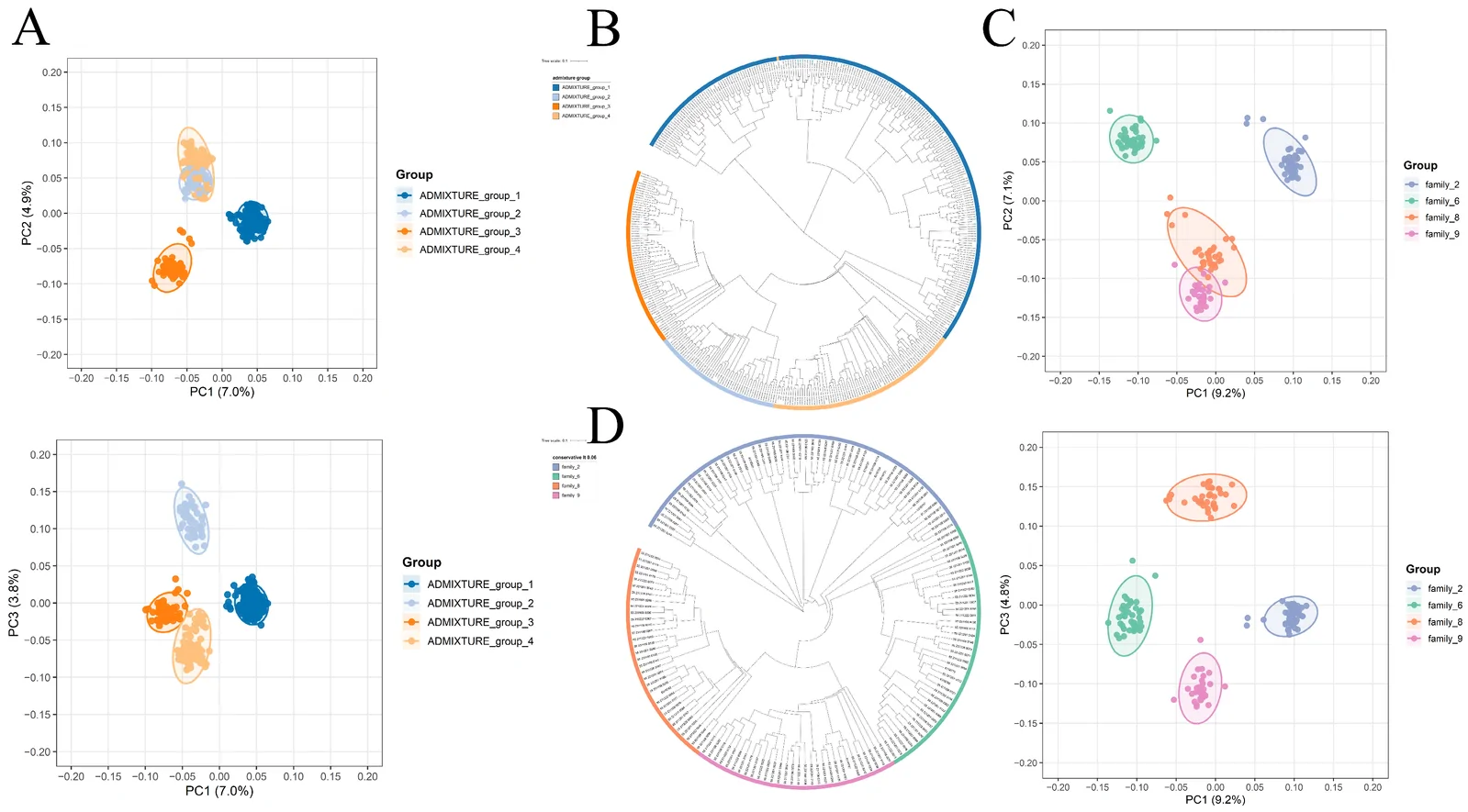
Genetic structure analyses of ADMIXTURE groups and genomic family groups. (**A**) PCA of ADMIXTURE groups. (**B**) NJ tree of ADMIXTURE groups based on Nei’s genetic distance. (**C**) PCA of genomic family groups. (**D**) NJ tree of genomic family groups based on Nei’s genetic distance. Ellipses in panels (**A**) and (**C**) indicate the 95% confidence regions for each group.

Based on the ADMIXTURE results, individuals with clear predominant ancestry proportion were assigned to four major genetic groups. PCA showed relatively clear separation among the four ADMIXTURE groups, with PC1, PC2, and PC3 explaining 7.0%, 4.9%, and 3.8% of the genetic variation, respectively (Figure 6A). PC1 and PC2 revealed the main pattern of genetic differentiation, with ADMIXTURE groups 1 and 3 forming distinct clusters separated from groups 2 and 4. Although groups 2 and 4 overlapped in the PC1-PC2 space, they were clearly separated along PC3 in the PC1-PC3 space. The NJ tree annotated by ADMIXTURE groups showed that individuals from the same group generally clustered on neighboring branches, further supporting the genetic differentiation reflected by the ADMIXTURE groups (Figure 6B).

Analysis based on genomic family group showed that families 2, 6, 8, and 9 formed relatively distinct clusters in PCA space, with PC1, PC2, and PC3 explaining 9.2%, 7.1%, and 4.8% of the genetic variation, respectively (Figure 6C). The NJ tree annotated by family groups also showed that individuals from the same family group generally clustered on neighboring branches (Figure 6D).

### 3.5. Contribution of Genetic Diversity Analysis

MAF distributions differed among the four ADMIXTURE groups (Figure S2). Group 1 had the most rare-allele SNPs (4423), whereas group 2 had the fewest (3020). In contrast, group 2 showed the largest numbers of SNPs in the 0.20–0.30 and 0.30–0.40 intervals.

We used ADMIXTURE-based clustering as the main classification scheme for evaluating group contributions to overall genetic diversity. The contribution of each group was quantified as the percentage loss or gain in total gene diversity and total allelic diversity after removing that group from the metapopulation. Both diversity measures were partitioned into within-subpopulation and between-subpopulation components.

**Figure 7:**
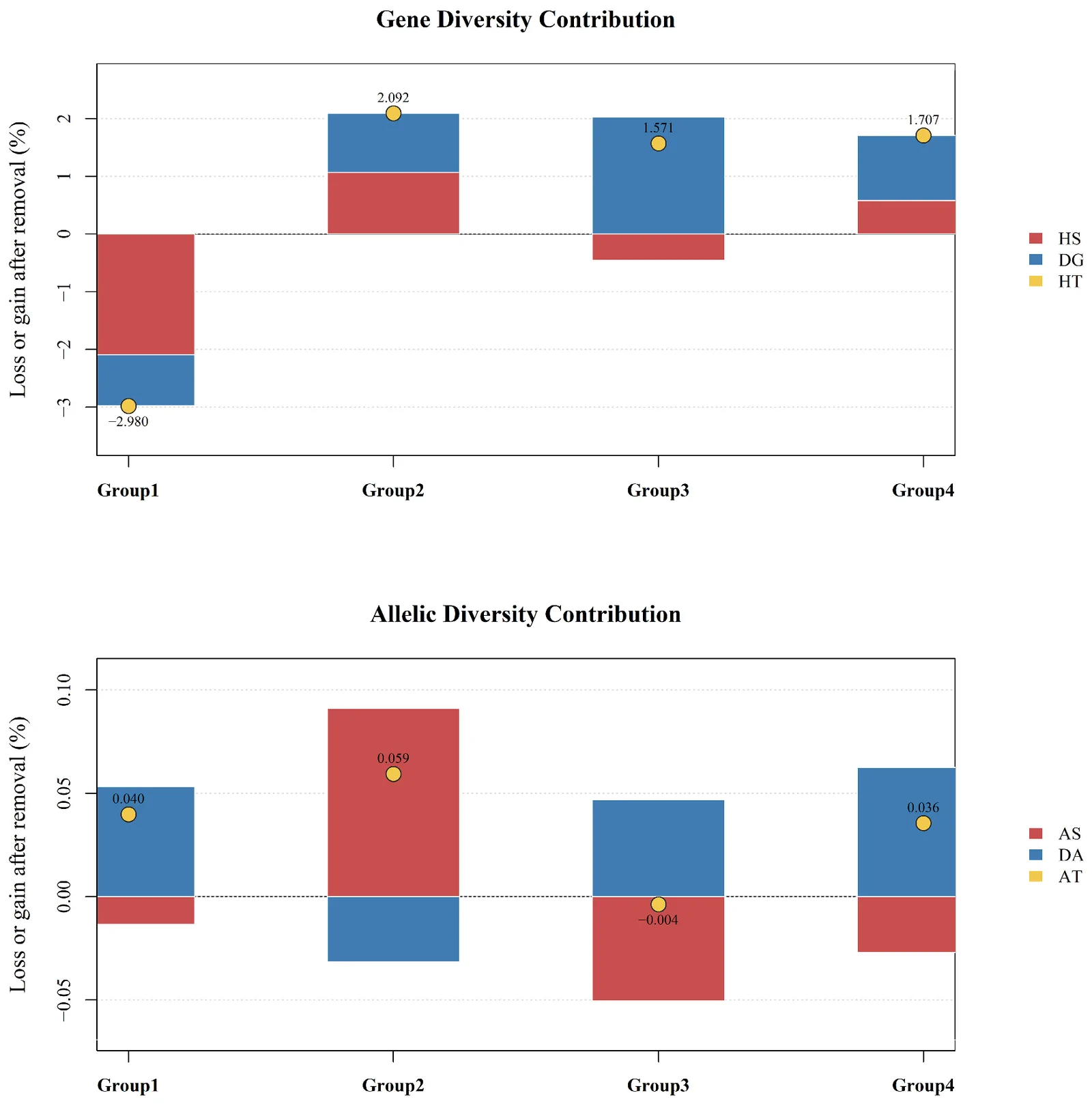
Contributions of ADMIXTURE groups to total gene and allelic diversity. Bars represent the loss or gain in total gene diversity (*H_T_*) and total allelic diversity (*A_T_*) after removing each group from the metapopulation. Positive total values indicate that the removal of a group leads to a decrease in the corresponding diversity component (i.e., the group contributes positively to diversity), whereas negative values indicate that removing the group increases the diversity measure.

The contributions of ADMIXTURE groups to gene and allelic diversity differed among groups. For gene diversity, groups 2, 3, and 4 showed positive contributions, with group 2 contributing the most (2.092%), whereas group 1 showed a negative contribution (Figure 7). For allelic diversity, group 1, 2, and 4 showed positive contributions, with group 2 again contributing the most (0.059%), whereas group 3 showed a slightly negative contribution. After Z-score standardization of the loss-or-gain contribution, group 2 showed the highest overall contribution to genetic diversity, followed by group 4. In contrast, group 3 and group 1 had negative final Z-scores (Table 6).

The optimal group proportions for maximizing gene and allelic diversity in synthetic pools (N = 1000), as well as their corresponding Z-score-standardized values, are presented in Table 7. For the pool maximizing gene diversity, group 2 showed the highest optimal proportion (50.3%), followed by group 4 (26.0%) and group 3 (20.1%), whereas group 1 had the lowest proportion (3.6%). In contrast, for the pool maximizing allelic diversity, Group1 showed the highest optimal proportion (54.8%), followed by group 4 (19.8%), group 3 (14.1%), and group 2 (11.3%). After Z-score standardization of the optimal synthetic-pool proportions, group 2 had the highest final Z-score (0.629), followed by group 1 (0.371).

## 4. Discussion

### 4.1. Distribution of Inbreeding Coefficients

Monitoring inbreeding is important for breeding decisions, slowing the accumulation of inbreeding, and managing conservation in small populations. Using 30K SNP array data, we evaluated genomic inbreeding in Japanese Brown cattle with multiple commonly used inbreeding estimators. Negative values were observed for all SNP-by-SNP inbreeding estimators except *F*_GRM05_, which is expected because these estimators can generally range from −1 to 1 for *F*_HOM_, from −1 to +∞ for *F*_GRM2_ and *F*_UNI_, and from −∞ to +∞ for *F*_GRM1_, respectively [6]. Compared with *F*_GRM1_, *F*_GRM2_ showed a broader distribution, a more positive tail, and more extreme negative values. This pattern is consistent with Villanueva et al. [59], as the marker-wise variance standardization used in VanRaden’s second method makes *F*_GRM2_ more sensitive to low-MAF loci than *F*_GRM1_. For most individuals carrying the common homozygous genotype at low-MAF loci, the standardized squared genotype deviation can be small. This lowers the average SNP-wise contribution and can generate more negative values. Conversely, a small number of individuals carrying low-frequency alleles at such loci may receive large positive contributions, producing a broader distribution and a positive tail. This mechanism may also explain the more compact distribution of *F*_GRM05_, because fixing allele frequencies at 0.5 gives all loci the same expected variance and avoids the disproportionate influence of low-MAF SNPs. Similarly, the distributional patterns of *F*_HOM_ and *F*_UNI_ may be partly related to their shared dependence on the allele frequency term $2p_{i}\left(1-p_{i}\right)$ in their calculations. *F*_GRM05_ produced the highest estimates in our dataset, consistent with a recent study of German Holstein cattle [60], probably because fixing allele frequencies at 0.5 defines a hypothetical base population with maximum expected heterozygosity and may inflate estimates when the true base-population frequencies deviate from 0.5 [61]. Among the ROH-based estimators, lowering the minimum number of consecutive homozygous SNPs progressively increased the estimates by detecting additional short ROH, while the numbers of segments longer than 8 Mb remained unchanged. This result is consistent with observations in Japanese Black cattle [34]. However, the additional short ROH detected under the less stringent settings in our dataset should be interpreted with caution. A recent comparison of medium- and high-density SNP panels in Holstein cattle found that some short ROH detected with the medium-density panel were not identified with the high-density panel, suggesting that they might represent artificial segments [62]. More generally, although *F*_ROH_ is independent of allele-frequency assumptions, its comparability across studies is limited by subjective ROH-calling criteria and differences in marker density, motivating the use of HBD-based estimators to quantify genome-wide autozygosity [38]. *F*_HBD_6R_ and *F*_HBD_7R_ showed nearly identical estimates and were closed to *F*_ROH_30_. Our corresponding means (0.150 and 0.151; 0.153) were slightly lower than those reported for Japanese Black cattle (0.162; 0.164), but the close agreement between HBD- and ROH-based estimates was consistent across the two breeds [34].

### 4.2. Relationship Among Inbreeding Coefficients

Pairwise Pearson correlations and PCA consistently separated the genomic inbreeding estimators into three broad groups. ROH- and HBD-based estimators formed the most concordant group (r = 0.96–1.00), indicating that they captured a shared signal of realized autozygosity despite differences in segment-definition criteria. *F*_GRM05_ was closely associated with this group, as shown by its high correlations with ROH/HBD-based estimators and its proximity to them in the PCA, suggesting that setting allele frequencies to 0.5 made it behave more like a general homozygosity measure than a conventional allele-frequency-dependent GRM coefficient. A similar result was also reported in Guzerá cattle [63], where *F*_ROH_ was more strongly correlated with both *F*_GRM05_ and *F*_HOM_ (r = 0.85 for both). *F*_GRM1_ and *F*_GRM2_ formed another distinct group, characterized by strong mutual correlation but clear PCA separation and weak or negative correlations with the ROH/HBD-*F*_GRM05_ group. Although *F*_UNI_ is theoretically a GRM-derived SNP-by-SNP estimator [59], it exhibited an intermediate pattern between the two groups. *F*_HOM_ formed a partially distinct third group: it remained closer to the ROH/HBD-*F*_GRM05_ group, consistent with its dependence on homozygosity, but was negatively correlated with *F*_GRM1_ and *F*_GRM2_ [64].

Caballero et al. [65] showed that ROH-based inbreeding coefficients can accurately estimate the rate of inbreeding depression in populations with low effective population size, whereas SNP-by-SNP estimators that give high weight to rare alleles may overestimate it. Therefore, in small and highly inbred Japanese Brown cattle populations, *F*_ROH_ may serve as a valuable indicator for population management, particularly when pedigree information is limited. Similarly, *F*_HBD_ may serve as a complementary indicator, as its comparable range, strong correlations, and close PCA clustering with ROH-based estimators suggesting a shared signal of realized autozygosity. It should be noted that the SNP-by-SNP estimators in our study were calculated using allele frequencies from the current population rather than from base population, which may make them work improperly [66]. Regarding this issue, Forutan et al. [61] showed that fixing allele frequencies at 0.5 for *F*_GRM_ yields high correlation with true inbreeding, which can be helpful when reliable base allele frequencies are unavailable. Given this reported correspondence, the close association of *F*_GRM05_ with *F*_ROH_ and *F*_HBD_ in our study provides additional support for the utility of ROH- and HBD-based estimators.

### 4.3. Population Structure and Genetic Diversity Analysis

Recent livestock studies have commonly used integrative analysis of PCA, phylogenetic tree, and ADMIXTURE to characterize population structure from complementary perspectives. These approaches visualize major genomic variation, summarize genetic relationships among populations, and infer latent ancestry components and admixture patterns [67,68,69,70]. Unlike previous studies where biogeography influenced population structure [58,67], our PCA analysis did not reveal clear regional differentiation (Figure 5). This finding may be due to the breeding history of the Kumamoto sub-breed of Japanese Brown cattle, in which breeding sires were exclusively developed within Kumamoto Prefecture and then spread across regions. A similar pattern was also reported in Holstein cattle by Salehi et al. [71], who found minimal genetic differentiation among four geographically distinct Holstein populations.

To further investigate population structure and provide a basis for population management, we evaluated the Kumamoto sub-breed of Japanese Brown cattle from two perspectives: (1) ancestry-based genetic structure inferred by ADMIXTURE with reference to breeding background information (K = 4), and (2) genomic family structure inferred from IBD relationships. Under each analytical framework, representative subpopulations selected using predefined criteria showed clear within-group clustering and between-group separation in PCA and NJ tree analyses. These results support the representativeness of the selected subpopulations and provide additional evidence that K = 4 is a reasonable level for describing the major genetic structure of this population. Since partial overlap was observed between groups 2 and 4, as well as between families 8 and 9, in the PC1–PC2 space (Figure 6A,C), we therefore examined the sire composition of these partially overlapping groups and found that some individuals assigned to different groups shared common sires, which may partially account for the observed overlap (Table 2 and Table 4). Notably, under both frameworks, two groups in each (groups 3 and 2; families 2 and 6) were predominantly composed of individuals descending from the two major sires: Shige-nami-izumi and Haru-yama-to/-sakae, implying the important role these two sires may have played in shaping the current population genetic structure. In addition, the major sires represented in ADMIXTURE group 1 and family 8 were paternal half-siblings, indicating similarity in their paternal backgrounds. However, ADMIXTURE group 4 and family 9 showed unrelated sire compositions. ADMIXTURE group 4 was mainly represented by Tsuru-tama (53.85%), which shared the same founder sire Mitsu-take with Mitsu-haru-shige, the main sire represented in ADMIXTURE group 1, whereas family 9 was dominated by Mitsu-shige-kuma-5 (76.7%), which originated from the founder sire Shige-nami. These results suggest that the correspondence between ADMIXTURE-based groups and IBD-based families was not always one-to-one, although both frameworks captured major paternal genetic components within the population. This discrepancy may partly arise because family assignments based on pairwise IBD kinship used more stringent thresholds, leading to finer subdivision compared with ADMIXTURE-based clustering.

Genetic diversity was another major concern in this study, as it builds a foundation for responding to environmental changes, disease outbreaks, and future breeding demands [72]. Both gene diversity (expected heterozygosity) and allelic diversity (allelic richness) have been incorporated into the genetic diversity assessment from a complementary perspective—the former reflecting short-term response to selection and adaptation, and the latter indicating long-term adaptive capacity [73,74]. Accordingly, conservation priorities based on these two diversity measures have been discussed in several livestock species, including pigs [75], goats [58], and chickens [76]. Beyond setting conservation priorities at the breed level, further stratification into lines may help define more specific conservation objectives within a breed [77]. In this study, to maximize sample utilization, the four ADMIXTURE groups (N = 429), representing the major genetic components of the population, were used as the primary units for evaluating gene and allelic diversity in Metapop2. Among the four ADMIXTURE groups, group 2 contributed most to both gene and allelic diversity and was ranked as the least inbred by most estimators (Table S3), supporting its potential for reducing inbreeding and improving genetic diversity. Conversely, group 1 showed the second-highest allelic diversity but the lowest gene diversity, while most homozygosity- and segment-based genomic inbreeding estimates ranked it as the most inbred group. This pattern suggests that group 1 may help retain rare alleles, but also exhibits substantial inbreeding, possibly related to the extensive use of its major sire, Mitsu-haru-shige. Therefore, group 1 should be regarded as a valuable but carefully managed genetic component, for which maintaining rare alleles while avoiding further increases in inbreeding should be prioritized. Accordingly, from a population management perspective, future breeding strategies should aim to gradually reduce dependence on the Mitsu-haru-shige lineage while moderately increasing the contribution of the group 2 paternal background represented by Haru-yama-to and Haru-yama-sakae.

In addition, we observed an inconsistency in priority rankings (final Z-score) between the loss-or-gain contribution results and the synthetic pool results: group 1 ranked last on the final Z-score from loss-or-gain contribution analysis, but ranked second in the synthetic pool analysis because of its relatively high contribution to allelic diversity (Table 6 and Table 7). A similar inconsistency was also observed in a study on Chinese indigenous goats, where Guizhou Black Goat (GZB) showed a negative *A_T_* contribution but a positive *D_A_* component in the loss-or-gain analysis, yet received the highest allocation proportion in the synthetic pool for maximizing allelic diversity [58]. This discrepancy likely reflects that some rare alleles occur at relatively higher frequencies in group 1, potentially favoring a larger synthetic-pool allocation to increase their probability of retention. However, because synthetic-pool optimization for allelic diversity is designed to maximize allele capture rather than to evaluate the overall contribution of a subpopulation to the existing metapopulation structure, the final Z-score derived from synthetic-pool proportions was considered as supporting evidence for the potential role of group 1 in allele retention rather than as the primary criterion for management decisions in our study.

## 5. Conclusions

This study compared ten genomic inbreeding estimators, assessed population structure, and evaluated genetic diversity contributions in the Kumamoto sub-breed of Japanese Brown cattle. ROH- and HBD-based estimators showed highly concordant patterns and may be useful indicators for monitoring realized autozygosity in this small population. Paternal background has played an important role in shaping the current genomic structure, as the representative groups were mainly associated with distinct major sire. ADMIXTURE group 2, mainly represented by the Haru-yama-to/-sakae sire background, may serve as a useful genetic resource for limiting future inbreeding accumulation and maintaining genetic diversity.

## Figures and Tables

**Table 1:** Summary statistics for the estimates of ten inbreeding coefficients.

Inbreeding Coefficient	Min	Max	Mean	Median	SD *
*F*_GRM1_	−0.206	0.289	−0.025	−0.038	0.097
*F*_GRM2_	−0.267	0.457	−0.033	−0.060	0.136
*F*_GRM05_	0.204	0.435	0.294	0.290	0.039
*F*_UNI_	−0.128	0.200	−0.033	−0.039	0.045
*F*_HOM_	−0.332	0.257	−0.033	−0.024	0.094
*F*_ROH_	0.041	0.313	0.132	0.126	0.045
*F*_ROH_30_	0.066	0.327	0.153	0.145	0.044
*F*_ROH_15_	0.104	0.361	0.197	0.191	0.043
*F*_HBD_6R_	0.071	0.336	0.150	0.144	0.041
*F*_HBD_7R_	0.073	0.336	0.151	0.145	0.040

* SD, standard deviation.

**Table 2:** ADMIXTURE groups and their major sire composition.

ADMIXTURE Group	*n*	Major Sire	Proportion
1	228	Mitsu-haru-shige	73.68%
2	52	Haru-yama-to Haru-yama-sakae	61.54%
3	71	Shige-nami-izumi	60.56%
4	78	Tsuru-tama	53.85%

**Table 3:** Number of retained genomic family groups and individuals under different between-family mean PI_HAT thresholds.

Between-Family PI_HAT Threshold	Number of Retained Family Groups	Number of Retained Individuals	Maximum Between-Group Mean PI_HAT
0.06	4	156	0.058
0.07	5	200	0.068
0.08	6	230	0.076
0.09	6	279	0.086
0.10	6	279	0.086

**Table 4:** Core genomic family groups and their major sire composition.

Family	*n*	Major Sire	Count	Proportion
Family 2	54	Shige-nami-izumi	41	75.90%
Family 6	39	Haru-yama-to	29	74.40%
Family 8	33	Taka-mitsu-shige	26	78.80%
Family 9	30	Mitsu-shige-kuma-5	23	76.70%

**Table 5:** Within- and between-family mean PI_HAT matrix of the four core families.

	Family 2	Family 6	Family 8	Family 9
Family 2	0.318			
Family 6	0.027	0.243		
Family 8	0.058	0.034	0.251	
Family 9	0.036	0.016	0.053	0.261

**Table 6:** Z-score ranking of ADMIXTURE groups based on the combined standardized contributions of total gene diversity (*H_T_*) and total allelic diversity (*A_T_*). Δ*H_T_* and Δ*A_T_* represent the percentage loss or gain in total gene diversity and total allelic diversity after removing each group from the metapopulation, respectively. To integrate both diversity components on a comparable scale, Δ*H_T_* and Δ*A_T_* were standardized as Z-scores and then summed to obtain the final score.

ADMIXTURE Group	ΔHT/%	ΔAT/%	Z-ΔHT	Z-ΔAT	Final Z-Score
Group2	2.092	0.059	0.624	1.008	1.632
Group4	1.707	0.036	0.463	0.105	0.568
Group3	1.571	−0.004	0.406	−1.379	−0.973
Group1	−2.98	0.04	−1.494	0.267	−1.226

**Table 7:** Z-score ranking of ADMIXTURE groups based on standardized optimal proportions in synthetic pools.

ADMIXTURE Group	Gene Diversity/%	Allelic Diversity/%	Z-Gene Diversity	Z-Allelic Diversity	Final Z-Score
Group2	50.3	11.3	1.308	−0.679	0.629
Group1	3.6	54.8	−1.106	1.477	0.371
Group4	26.0	19.8	0.052	−0.258	−0.206
Group3	20.1	14.1	−0.253	−0.540	−0.793

## Data Availability

The original contributions presented in this study are included in the article/Supplementary Material. Further inquiries can be directed to the corresponding authors.

## Supplementary Materials

The following supporting information can be downloaded at: https://www.mdpi.com/article/10.3390/ani16162587/s1: Figure S1: Sampling locations and rearing regions; Figure S2: MAF distribution among ADMIXTURE groups; Table S1: ADMIXTURE fivefold cross-validation errors from K2 to K20; Table S2: Within- and between-family mean PI_HAT values and family sizes of candidate genomic family groups; Table S3: Mean genomic inbreeding coefficients by ADMIXTURE group.

## Abbreviations

The following abbreviations are used in this manuscript:

SNP	Single-nucleotide polymorphism
PCA	Principal component analysis
NJ	Neighbor-joining
ROH	Runs of homozygosity
HBD	Homozygosity by descent
AI	Artificial insemination
ETA	Estimated transmitting ability
BMS No.	Beef Marbling Standard number
IBD	Identity by descent
NLBC	National Livestock Breeding Center
MAF	Minor allele frequency
GRM	Genomic relationship matrix
HMM	Hidden Markov model
LD	Linkage disequilibrium
*H_T_*	Total gene diversity
*H_S_*	within-subpopulation component of gene diversity
*D_G_*	between-subpopulation component of gene diversity
*A_T_*	Total allelic diversity
*A_S_*	average within-subpopulation allelic diversity
*D_A_*	Between-subpopulation component of allelic diversity
SD	Standard deviation
PC	Principal component
